# The diagnostic value of serum creatinine and cystatin c in evaluating glomerular filtration rate in patients with chronic kidney disease: a systematic literature review and meta-analysis

**DOI:** 10.18632/oncotarget.20271

**Published:** 2017-08-16

**Authors:** Xilian Qiu, Chunyong Liu, Yuqiu Ye, Huiqun Li, Yanbing Chen, Yongmei Fu, Zhenjie Liu, Xianzhang Huang, Yunqiang Zhang, Xueyuan Liao, Hongyong Liu, Wenbo Zhao, Xun Liu

**Affiliations:** ^1^ Department of Laboratory Medicine, The First Affiliated Hospital of Sun Yat-sen University, Guangzhou, China; ^2^ Department of Laboratory Medicine, The Second Affiliated Hospital of Guangzhou University of Traditional Chinese Medicine, Guangzhou, China; ^3^ Department of Nephrology, The Third Affiliated Hospital of Sun Yat-sen University, Guangzhou, China; ^4^ Medical genetic center, Guangdong Women And Children Hospital, Guangzhou, Guangdong, China; ^5^ Division of Nephrology, The 3rd Affiliated Hospital of Sun Yat-sen University, Yuedong Hospital, Meizhou, China

**Keywords:** creatinine, cystatin C, glomerular filtration rate, meta-analysis

## Abstract

**Background:**

Serum biomarkers, such as serum creatinine (SCr) and serum cystatin C (SCysC), have been widely used to evaluate renal function in patients who have chronic kidney disease (CKD).

**Objective:**

This article aims to assess the value of determining SCr and SCysC levels in patients that have long-term kidney disease. Approaches: MEDLINE, EmBase, the Cochrane Library and other databases were searched using both MeSH terms and text words to collect research that assessed the diagnostic value of using SCr and SCysC to evaluate Glomerular Filtration Rate (GFR) in patients with CKD. Data were converted into fourfold tables. Summary Receiver Operating Characteristic Curves and meta-analyses were accomplished via Meta-Disc version 1.4.

**Results:**

In total, 21 relevant articles involving 3112 study subjects were included in our review. Results showed that the collective sensitivity for SCr and SCysC was 0.77 (95% CI: 0.69–0.84) and 0.87 (95% CI: 0.82–0.91), respectively. The pooled specificity for SCr and SCysC was 0.91 (95% CI: 0.86–0.94) and 0.87 (95% CI: 0.82–0.91), respectively. Subgroup analyses demonstrated that when GFR cut-off values are set to 60 (ml/min/1.73 m^2^), the pooled sensitivity is 0.94 (95% CI: 0.90–0.96) for SCysC and 0.75 (95% CI: 0.68–0.82) for SCr.

**Conclusions:**

The diagnostical accuracy for impaired kidney function favors SCysC. Confidence intervals for the pooled sensitivity and specificity for SCr and SCysC overlap. However, SCysC is more sensitive for estimating GFR than SCr when GFR cut-off values are set to 60 (ml/min/1.73 m^2^).

## INTRODUCTION

CKD, as well as end stage renal disease (ESRD), presents serious risks to human health. Worldwide, the incidence and prevalence of CKD dramatically increases with aging [1, 2]. As a progressive disease, CKD in many cases leads to ESRD. Accurate and convenient evaluation of renal function is important for both healthy populations and patients with CKD, especially those with mild to moderate decreased renal function. Early initiation of treatment in CKD patients has shown that it is possible to delay or even prevent the frequency and severity of adverse outcomes. Hence, early stage prognosis of CKD is required for early initiation of treatment to help patients, in particular, those at greatest risk for progression. GFR is regarded as a significant indicator for kidney surgery, and this measurement is considered to be the gold standard for evaluating renal disease. In addition, GFR is utilized as an independent risk factor affecting cardiovascular function [3]. It was found that a low GFR is associated with increased mortality, cardiovascular events and hospitalizations. Hence, GFR acts as an important indicator in the diagnosis of patients, as well as in clinical management.

In addition to SCysC, urine sediment abnormalities, and albuminuria, SCr has also been used as a traditional endogenous biomarker for renal function. Researchers commonly utilize the clearance rate of exogenous filtration markers in evaluating GFR, including radioactive materials such as inulin, ^125^I-iothalamate, iohexol, ^99m^Tc-DTPA, ^51^Cr-EDTA, among others. Although incompatible with routine monitoring, experts use these materials in professional research and even in clinical trials [4–7].

Estimating GFR is a method used to measure endogenous substances in the blood. SCr is indicated in many studies as having less sensitivity for kidney failure, especially in patients with minor kidney dysfunction and in older CKD patients in whom kidney deficiency is often under-recognized. Recently, in accordance with these new findings, SCysC has been proposed as a filtration marker of GFR to replace SCr. Many studies [8–11] recently demonstrated that SCysC is a more sensitive serum marker than SCr for evaluating renal glomerular filtration function damage; however, there are some differing points of view [12]. To the best of our knowledge, only Van Pottelbergh, et al. [13] completed a systematic review in 2010 that tried to investigate and ascertain the best process to provide measurements of renal function. However, they could not clearly define which methods were most accurate at evaluating kidney function in people with CKD as they examined only a small number of prospective studies and populations, and only included articles written in English. There has been no update until 2010, when several groups compared the Modification of Diet in Renal Disease (MDRD) and Cockcroft-Gault (CG) formulas and other equations, such as the Chronic Kidney Disease Epidemiology Collaboration (CKD-EPI) equation [14–18]. Therefore, an updated heterogeneous meta-analysis including the Chinese population is needed to better evaluate kidney function.

## MATERIALS AND METHODS

### Literature search strategy

We undertook a systematic meta-analysis in accordance with Preferred Reporting Items for Systematic Reviews and Meta-analyses (PRISMA) guidelines (see Supplementary Table 2) and the Standards for Reporting of Diagnostic Accuracy (STARD) guidelines. We searched population-based studies and different cross-sectional studies that included patients with CKD. In detail, we searched MEDLINE (via PubMed), the Cochrane Library, EmBase, Chinese National Knowledge Infrastructure and the Chinese Biomedical Literature Database, as well as other databases and relevant conference meetings from inception to 13th, Feb. 2017, using the following keywords and corresponding medical subject heading: *“Chronic* kidney disease”, “CKD”, “end stage renal disease”, “ESRD”, “cystatin C”, “SCysC”, “creatinine”, “SCr”, “glomerular filtration rate”, “GFR””, “diagnosis test”, “sensitivity”, “specificity”, and synonyms. In the search strategy, subject terms and keywords were applied in combination. Searches using other engines such as Google Scholar on the internet were performed in supplement. No restriction was placed on language or publication forms. Authors of resource studies were contacted by email and telephone for help if examined reports lacked details or enough applicable information.

Two independent reviewers (Chunyong Liu and Huiqun Li) evaluated the articles, and screened titles and abstracts to assess eligibility and remove duplicates. Discrepancies were resolved by discussion among all authors. Search strategies of all databases could be found in Supplementary Table 1.

### Study selection criteria

We excluded studies as follows: publications containing the same information; studies with inadequate facts; personal opinions, meetings, reviews and meta-analysis articles; animal and cell studies; studies including fewer than 30 people. Diagnostic randomized controlled trials (D-RCT) were also not included. Studies were considered suitable for inclusion if they met criteria including: (a) studies investigating the use of SCr and SCysC when calculating GFR in patients with CKD; (b) studies providing statistical data such as extracted population characteristics, total size of research conducted, sensitivity and specificity, cut-off values, true and false positive and negative data or if such data could be extracted by reading context; (c) the search attempted to identify diagnostic accuracy test (DAT); (d) the use of gold standard tests, including measurements of inulin, ^125^I-iothalamate, ^99m^Tc-DTPA, iohexo和l, ^51^Cr-EDTA [19–21].

### Detection method

For SCr and SCysC detection, all clinical methods were included. The testing processes for SCysC were particle-enhanced nephelometric immunoassay (PENIA) and particle-enhanced turbidimetric immunoassay (PETIA) [22, 23]. The detection methods for SCr were the enzymatic method and the Jaffé method [24].

### Data extraction and literature quality assessment

Independent reviews by Chunyong Liu and Huiqun Li highlighted other possible studies in accordance with the above described exploration approach. Both reviews and summaries of every article were studied repeatedly to be sure inclusion criteria were met.

In cases of several publications focusing on the same research, we included the one with the largest and most detailed data only. Any disagreement between the two reviewers was resolved with the help of a third reviewer (Xilian Qiu).

Data were extracted in accordance with a standardized form, and controversies surrounding differing data were resolved by consensus. Extracted study characteristics were country of origin, year of publication, study size, age, gender prevalence of CKD, kind of risk and adjusted confounding factors. Summary specificity (SPE), summary sensitivity (SEN), summary positive and summary negative predictive values (± PVs), summary positive and summary negative likelihood ratios (± LRs), summary receiver operating characteristic (SROC) curves, area under the summary receiver operating characteristic (SAUC), and diagnostic tests combined odds ratio (DOR) were also determined. One author (Chunyong Liu) entered the data separately into the software RevMan version 5.3, Stata software version 14.0 and Meta-Disc version 1.4. A second author (Huiqun Li) and a third author (Xilian Qiu) independently checked the data entry.

Two investigators (Chunyong Liu and Huiqun Li) independently evaluated the quality of studies in this meta-analysis via the quality evaluation of diagnostic accuracy studies-2 (QUADAS-2) system, which was described by Whiting, P. [25, 26]. Criteria consisted of four components (patient selection, index test, reference standard, flow and timing), and was analyzed by the RevMan 5.3 software. Each component was evaluated for risk of bias, and the first three components are also evaluated in terms of applicability. Signaling questions were involved to help judge the risk of bias. The quality assessment of the involved studies was independently carried out by Chunyong Liu and Huiqun Li.

### Synthesis and analysis of data

Meta-analysis was carried out by the software RevMan version 5.3, and Meta-Disc version 1.4. Publication bias analysis was performed using Stata 14.0 (Stata Corp, College Station, TX, USA). When a fourfold table contained a cell with a value of 0, the calculations were corrected with the addition of 0.5 in the cell. Studies containing two cells with the value of 0 were not included in the analysis [26]. Each document was summarized by SPE, SEN, ± PVs, ± LRs for diagnostic tests, and also analyzed for heterogeneity with the χ^2^ test. This was evaluated using the I^2^ method, and significant study heterogeneity was considered when I^2^ > 50%. I^2^ values between 25 and 50% were deemed to show modest heterogeneity and I^2^ values < 25% were thought to indicate low heterogeneity. Clinical utility of SCysC and SCr for estimated GFR (eGFR) was evaluated by a Fagan plot. We drew the SROC curve based on the literature included in this study, and calculated the zone under the receiver operating characteristic curves. All results are presented with 95% confidence intervals (CI) [27].

## RESULTS

### Characteristics of the literature search and screening

**Figure 1:**
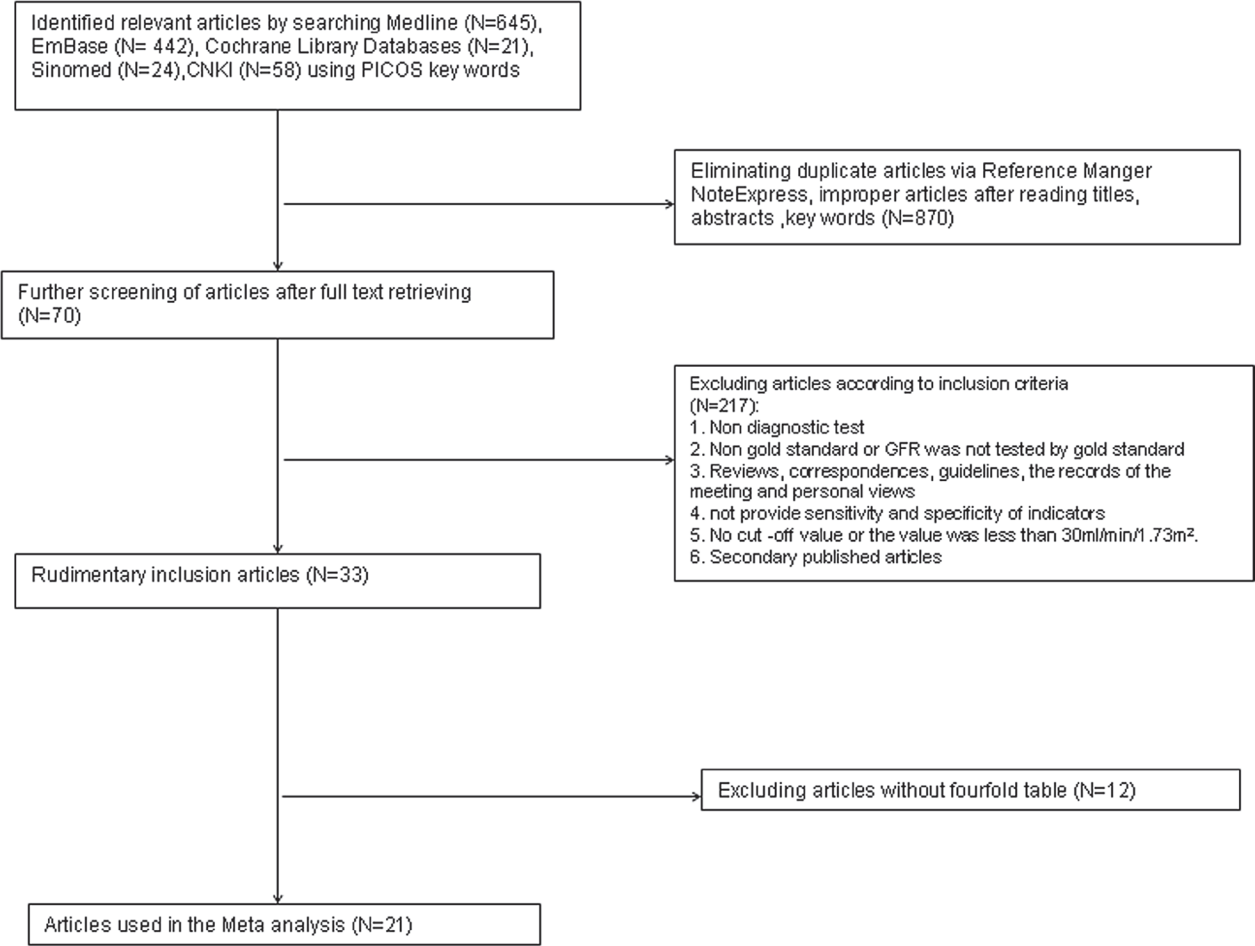
Flowchart representing study selection for systematic review of SCr and SCysC as a diagnostic tool for renal CKD evaluation

A flow chart of the literature search and literature screening created using RevMan 5.3 is shown clearly in Figure 1. The original literature search identified 1190 studies. Of these, 1097 studies were excluded due to duplication or lack of accordance with inclusion criteria, and 70 studies were eliminated after reading titles and abstracts carefully. In the end, only 33 publications met the inclusion criteria. We tried to contact 11 corresponding authors of 12 studies to obtain details for the fourfold table or to complete information, but were unable to make contact or obtain useful information. Finally, 12 publications were eliminated due to incomplete fourfold table information, and 21 publications were used for the present research. Among these, 17 were published in English, and four were written in Chinese. All studies focused on the diagnostic test for GFR values via SCr and SCysC.

General information was extracted from 21 literature sources comprising 3112 patient cases. The number of males was generally slightly higher than females. For the assessment of renal function, the cut-off values of SCysC and SCr were 0.81–1.74 mg/L and 75.1–149.0 μmol/L, respectively. Four literature sources made no reference to these indicators. The cut-off value of measured GFR tested by the gold standard was 60–90 ml/min/1.73m^2^. The main characteristics of the literature are shown in Table 1 and Table 2.

**Table 1:** Main characteristics of 21 studies selected for a meta-analysis of the diagnostic value of SCr and SCysC in the evaluation of GFR

Author Year	Country of residence	Study Participants NO. of cases/NO of patients	Average age	Male (%)	Golden Stanard	Detection method of Cys C	Detection method of Cr
Burkhardt, H 2002 [41]	Germany	30	75.4	50	Inulin clearance	Cys C PETIA assay	Cr Jaffé method
Chantrel, F 2000 [42]	France	161	39	49.1	inulin	Cys C PENIA assay	Cr Enzyme method
Donadio, C 2010 [43]	Italy	295	52.4	53.6	99mTc-DTPA	Cys C PENIA assay	Cr Jaffé method
Donadio, C 2012 [44]	Italy	367	52.7	46.9	99mTc-DTPA	PENIA & PETIA assay	Cr Jaffé method
Filler, G 2002 [45]	Canada/Germany	225	/	59.5	51Cr-EDTA	Cys C PENIA assay	Cr Enzyme method
Funda Aydin 2015 [46]	Turkey	84	61	70.2	99mTc-DTPA	PENIA assay	Cr Jaffé method
Harmoinen, A P 1999 [47]	Finland	51	/	51	51Cr-EDTA	Cys C PETIA assay	/
Hojs, R 2006 [48]	Slovenia	164	57.5	52.4	51Cr-EDTA	Immunonephelometric assay	Cr Jaffé method
Kyhse-Andersen, J 1994 [49]	Denmark/Sweden	51	/	53	/	Cys C PETIA assay	Cr Enzyme method
Li Hai-xia 2005 [50]	China	51	54	49	99m Tc-DTPA	Cys C PENIA assay	Cr Jaffé method
Li Hai-xia 2006 [51]	China	265	53	55.5	99m Tc-DTPA	Cys C PENIA assay	Cr Jaffé method
Li Ping 2005 [52]	China	71	/	/	99m Tc-DTPA	Cys C PETIA assay	Cr Jaffé method
Macisaac, R J 2007 [53]	Australia	251	60	61	99mTc-DTPA	Cys C PENIA assay	Cr Jaffé method
Newman, D J 1995 [54]	Sweden	206	/	55.8	51Cr-EDTA	Cys C PETIA assay	Cr Jaffé method
Pöge, U 2006 [55]	Germany	105	49.5	58.1	99mTc-DTPA	Immunonephelometric assay	Cr Jaffé method
Rigalleau, V 2008 [56]	France	124	62	62.9	51Cr-EDTA	Cys C PENIA assay	Cr Jaffé method
Spanaus, K S 2010 [57]	Germany, Austria, Italy	227	45.7	67.8	iohexol	Cys C PENIA assay	Cr Jaffé method
Stickle, D 1998 [58]	USA	34	/	NA	inulin clearance	Cys C PETIA assay	picric acid assay
Van Den Noortgate, N J 2002 [59]	Belgium	48	84.4	35	51Cr-EDTA	Cys C PETIA assay	Cr Jaffé method
Wang Hong-ru 2010 [60]	China	76	55. 01	44.7	99m Tc-DTPA	Cys C PETIA assay	Cr Enzyme method
Zhou You 2008 [61]	China	186	56. 13	53.2	99m Tc-DTPA	Cys C PENIA assay	Cr Jaffé method

CKD = chronic kidney disease; SCr = serum creatinine; SCysC = serum cystatin C; GFR = glomerular filtration rate; mGFR = measured GFR; PETIA = particle-enhanced turbidimetric immunoassay; PENIA = particle-enhanced nephelometric immunoassay; NA or “/”= not applicable.

**Table 2:** The cut-off value of GFR, SCysC, SCr, fourfold table data, sensitivity and specificity index of the adopted literature

Author Year	The cut-off value of GFR (mL/min/1.73 m^2^)	Cut-off values		TP	FP	FN	TN	r	Sensitivity (%)	Specificity (%)
Burkhardt, H 2002 [41]	70	Cystatin C	1.63	7	5	5	13	/	0.58	0.72
		Serum creatinine	82	11	7	1	11	/	0.92	0.61
Chantrel, F 2000 [42]	90	Cystatin C	0.9	58	7	19	77	0.7	0.75	0.92
		Serum creatinine	1.1	55	5	22	79	0.74	0.71	0.94
Donadio, C 2010 [43]	90	Cystatin C	0.95	225	3	39	28	0.93	0.85	0.9
		Serum creatinine	105	/	/	/	/	/	/	/
Donadio, C 2012 A [44]	90	Cystatin C	0.95	222	1	6	28	0.91	0.66	0.97
		Serum creatinine	105	219	1	9	28	0.94	64.7	96.5
Funda Aydin 2015 [46]	90	Cystatin C	1.02	33	11	2	38	0.90	0.93	0.78
		Serum creatinine	118	39	6	6	33	0.80	0.86	0.85
Filler, G 2002 [45]	90	Cystatin C	1.11	60	8	15	142	−0.76	0.8	0.95
		Serum creatinine	83	26	8	49	142	−0.5	0.35	0.95
Harmoinen, A P 1999 [47]	80	Cystatin C	1.2	14	6	3	28	0.77	0.82	0.82
		Serum creatinine	104	6	2	10	33	0.56	0.038	0.94
Hojs, R 2006 [48]	60	Cystatin C	1.74	67	22	4	71	0.79	0.95	0.77
		Serum creatinine	149	60	6	26	72	/	0.7	0.92
Kyhse-Andersen, J 1994 [49]	80	Cystatin C	/	19	2	5	25	/	0.79	0.93
		Serum creatinine	/	14	2	10	25	/	0.58	0.93
Li Hai-xia 2005 [50]	80	Cystatin C	1	13	14	1	23	0.74	0.93	0.62
		Serum creatinine	113	12	15	2	22	0.65	0.48	0.96
Li Hai-xia 2006 [51]	80	Cystatin C	1.17	163	38	12	52	0.66	0.93	0.58
		Serum creatinine	107	140	12	35	78	0.53	0.8	0.87
Li Ping 2005 [52]	80	Cystatin C	1.43	48	2	3	18	0.9	0.94	0.9
		Serum creatinine	112	46	4	5	16	0.85	0.92	0.8
Macisaac, R J 2007 [53]	60	Cystatin C	0.81	53	20	1	177	0.81	0.98	0.9
		Serum creatinine	90	54	26	11	161	0.72	0.83	0.86
Newman, D J 1995 [54]	72	Cystatin C	1.25	60	6	24	116	0.81	0.71	0.95
		Serum creatinine	110	44	10	40	112	0.5	0.52	0.92
Pöge, U 2006 [55]	60	Cystatin C	1.41	84	1	7	13	0.86	0.92	0.93
		Serum creatinine	/	/	/	/	/	/	/	/
Rigalleau, V 2008 [56]	60	Cystatin C	1.56	70	6	6	42	0.82	0.93	0.88
		Serum creatinine	148	/	/	/	/	0.79	/	/
Spanaus, K S 2010 [57]	90	Cystatin C	0.91	147	25	8	47	0.87	0.95	0.76
		Serum creatinine	91	143	26	12	46	0.85	0.92	0.64
Stickle, D 1998 [58]	90	Cystatin C	1.4	20	0	3	11	0.87	0.87	1
		Serum creatinine	79.5	21	1	2	10	0.9	0.91	0.9
Van Den Noortgate, N J 2002 [59]	80	Cystatin C	1.09	28	0	11	9	0.62	0.72	1
		Serum creatinine	75.1	22	0	17	9	0.68	0.56	1
Wang Hong-ru 2010 [60]	90	Cystatin C	1.35	44	2	18	12	−0.81	0.71	0.86
		Serum creatinine	/	/	/	/	/	−0.73	/	/
Zhou You 2008 [61]	80	Cystatin C	/	114	27	9	36	−0.66	0.96	0.57
		Serum creatinine	/	99	8	24	55	−0.52	0.8	0.87

### Quality assessment of the included literature

**Figure 2:**
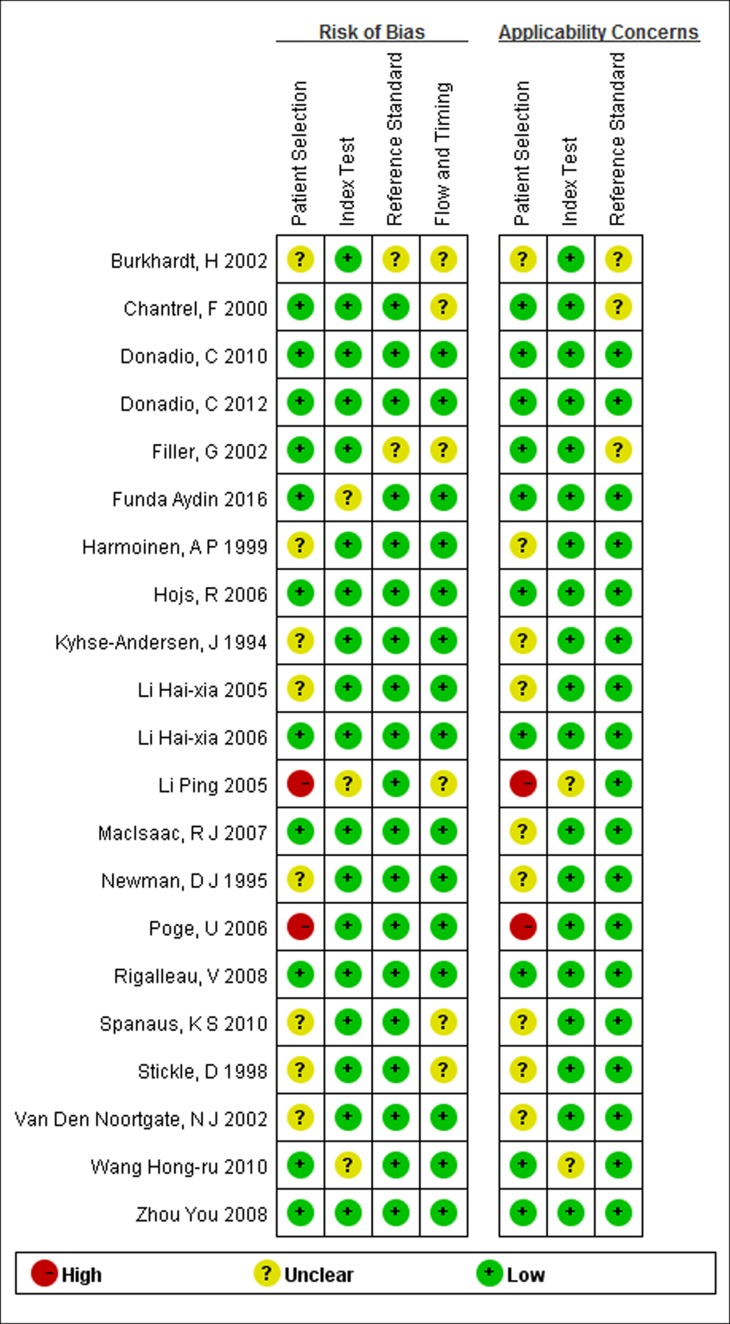
Results of the evaluation of each study according to QUADAS-2 See the colors in the online version.

**Figure 3:**
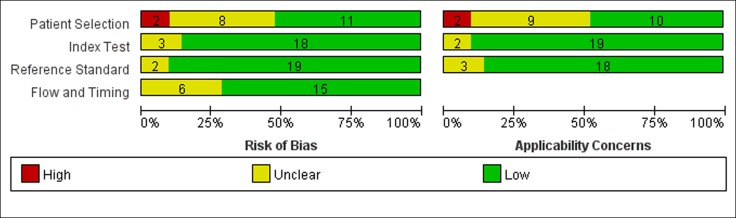
Methodological quality assessment summary by QUADAS-2

QUADAS-2 results are shown in Figure 2 and Figure 3. Regarding the risk of bias, there were six studies that fulfilled all QUADAS-2 criteria. In eight studies, there was uncertain risk of bias in patient selection, and two studies showed a high risk of bias in patient selection. Three studies showed uncertain risk of bias in the index test, and two studies showed unclear risk of bias in the reference standard. Five studies showed uncertain risk of bias in the flow and timing. Some studies [16, 18, 22, 23, 25] enrolled mostly female or male patients, who might not be representative of the target population, and so potential spectrum bias existed.

The results of the quality assessment for the 21 included studies are shown in Figure 2. Generally, most of the included studies met the quality criteria.

Figure 3 shows the integration of the methodological quality of included papers based on the foundation of reviewers’ assessments with regard to the four areas evaluating the risk of bias and the three domains evaluating applicability issues for the QUADAS-2 checklist for each study. Specifically, studies with a small bias risk or small applicability issues are highlighted in green and the studies with a high risk of applicability or bias issues are in red. Studies where risks of bias or applicability issues could not be assessed properly are indicated in yellow.

### Meta-analysis results

All included studies investigated the diagnostic accuracy of both SCr and SCysC in predicting CKD diseases. In total, 21 studies were included and 17 of them provided complete data for this meta-analysis. Regarding the diagnostic 2 × 2 table, all 21 studies provided the value of SCysC, while 17 studies provided the value of SCr.

**Figure 4:**
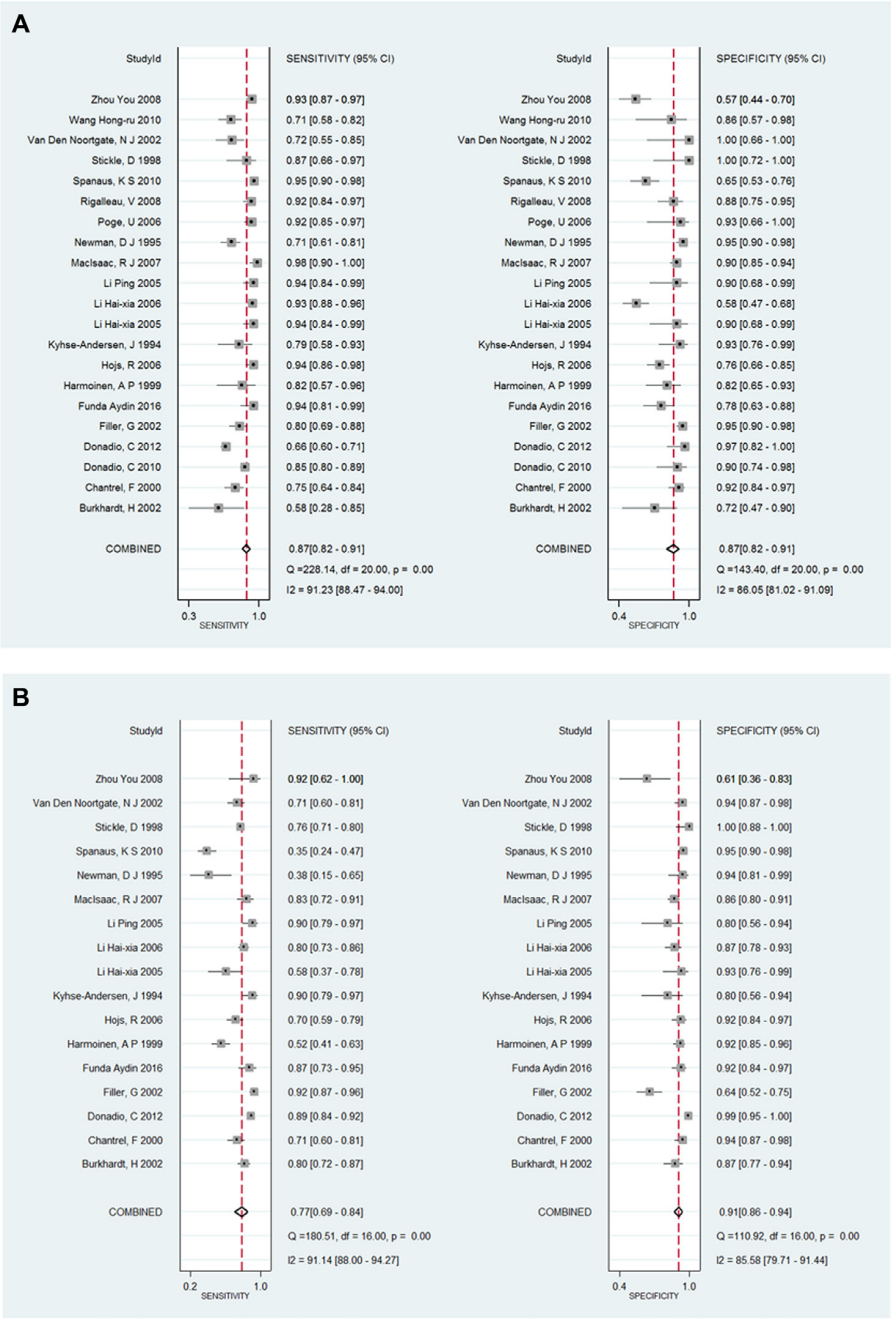
(**A**) The forest plot of sensitivity and specificity for SCysC in evaluating eGFR. (**B)** The forest plot of sensitivity and specificity for SCr in evaluating eGFR.

The pooled diagnostic accuracy of SCysC and SCr were tested. Across all settings, the pooled sensitivity for SCr and SCysC was 0.77 (95% CI: 0.69–0.84) and 0.87 (95% CI: 0.82–0.91), respectively, and the pooled specificity for SCr and SCysC was 0.91 (95% CI: 0.86–0.94) and 0.87 (95% CI: 0.82–0.91), respectively. CIs for pooled sensitivity and specificity for SCr and SCysC overlap. Analysis results of SCysC and SCr did not show that SCysC had the highest pooled sensitivity and specificity. Figure 4A and Figure 4B below shows the sensitivity and specificity of SCysC and SCr, respectively. Results for the forest plot of positive LR and negative LR, the forest plot of the DOR in evaluating eGFR, and the SROC Curve for SCr and SCysC are shown from Supplementary Figure 1 to Supplementary Figure 9.

### Subgroup analysis

**Figure 5:**
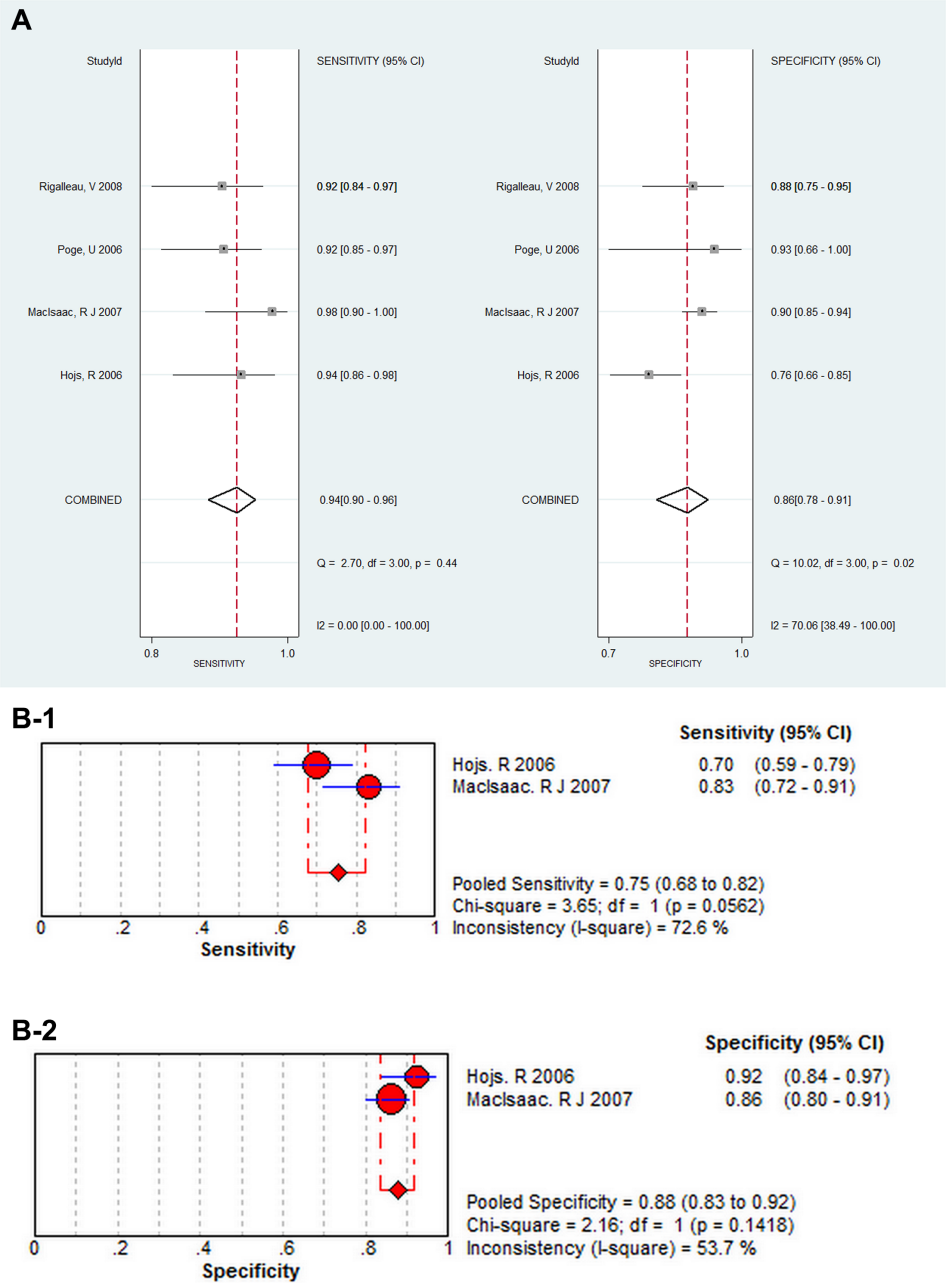
(**A**) The subgroup analysis for SCysC when cut-off values as 60 (mL/min/1.73 m^2^), the forest plot of sensitivity and specificity for SCysC in evaluating eGFR. (**B-1)** The subgroup analysis for SCr when cut-off values as 60 (mL/min/1.73 m^2^), the forest plot of sensitivity for SCr in evaluating eGFR. (**B-2)** The subgroup analysis for SCr when cut-off values as 60 (mL/min/1.73 m^2^), the forest plot of specificity for SCr in evaluating eGFR.

**Figure 6:**
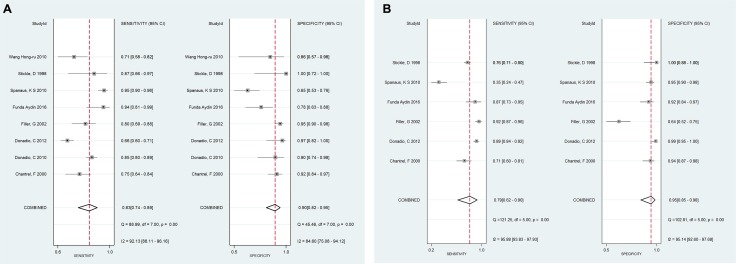
(**A**) The subgroup analysis for SCysC when cut-off values as 90 (mL/min/1.73 m^2^), the forest plot of sensitivity and specificity for SCysC in evaluating eGFR. (**B** )The subgroup analysis for SCr when cut-off values as 90 (mL/min/1.73 m^2^), the forest plot of sensitivity and specificity for SCr in evaluating eGFR.

The pooled diagnostic accuracy of SCysC and SCr using different GFR cut-off values of 60 (ml/min/1.73 m^2^) and 90 (ml/min/1.73 m^2^) were tested. GFR cut-off values of 60 (ml/min/1.73 m^2^), from Figure 5A, revealed a pooled sensitivity for SCysC of 0.94 (95% CI: 0.90–0.96), and a pooled specificity for SCysC of 0.86 (95% CI: 0.78–0.91). Using GFR cut-off values of 60 (ml/min/1.73 m^2^), shown in Figure 5B, pooled sensitivity for SCr was 0.75 (95% CI: 0.68–0.82), and pooled specificity for SCr was 0.88 (95% CI: 0.83–0.92). Diagnostic accuracy of impaired renal function favors SCysC. SCysC is more sensitive for estimating GFR than SCr when the GFR cut-off values are set as 60 (ml/min/1.73 m^2^). The GFR cut-off values of 90 (ml/min/1.73 m^2^), from Figure 6A, resulted in a pooled sensitivity for SCysC of 0.83 (95% CI: 0.74–0.89) and a pooled specificity for SCysC of 0.90 (95% CI: 0.82–0.95). From Figure 6B, pooled sensitivity for SCr was 0.79 (95% CI: 0.62–0.90), and pooled specificity for SCr was 0.95 (95% CI: 0.85–0.98).

### Heterogeneity analysis and publication bias analysis

**Figure 7:**
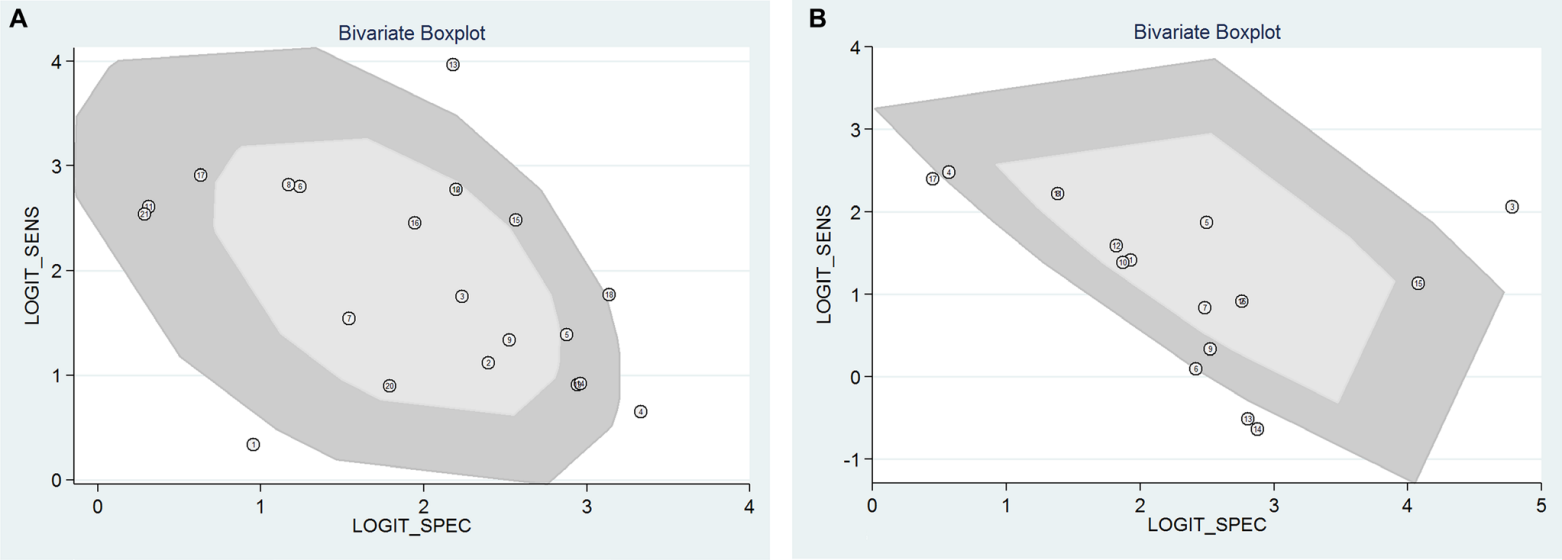
(**A**) Bivariate boxplot heterogeneity analysis of the SCysC detection; (**B)** Bivariate boxplot heterogeneity analysis of the SCr detection.

**Figure 8:**
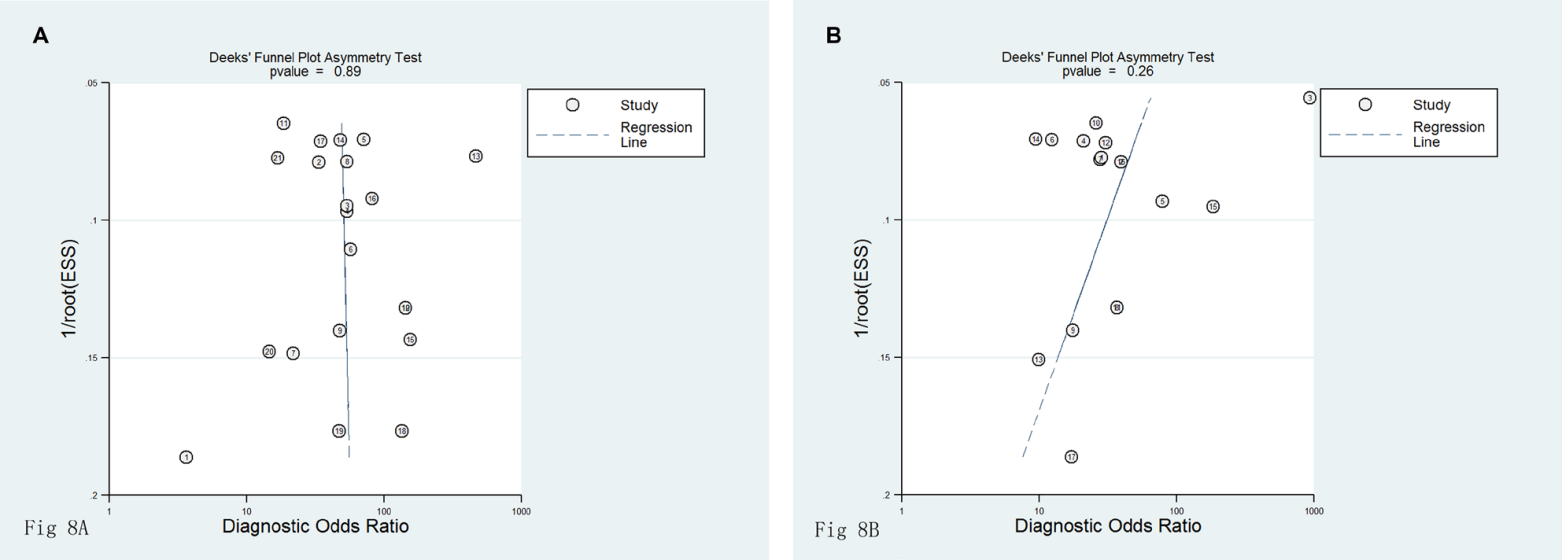
(**A**) Funnel plot of publication biasSCysC detection of studies included. (**B)** Funnel plot of publication bias for creatinine detection of studies included.

Heterogeneity analysis values for each research publication are displayed in Figure 7A and Figure 7B. Heterogeneity analysis regarding SCr and SCysC detection showed that there was no heterogeneity for most studies. Deeks funnel plot asymmetry test for all examinations showed no publication bias. For SCysC detection, *P* = 0.89 (Figure 8A), and for SCr detection, *P* = 0.26 (Figure 8B).

### SROC curves

**Figure 9:**
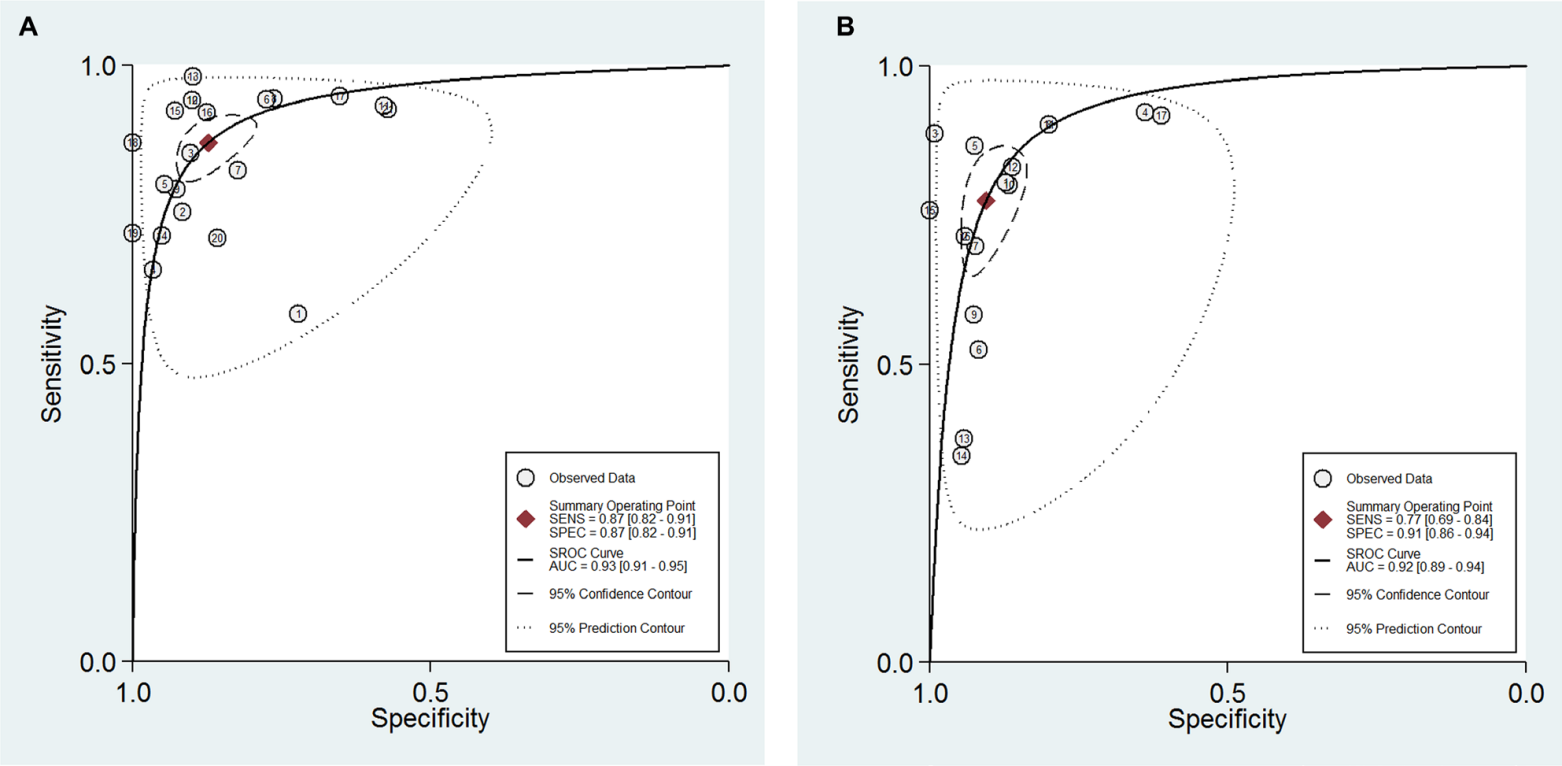
(**A**) SROC curve of the diagnostic value of GFR via SCysC. (**B)** SROC curve of the diagnostic value of GFR via SCr.

**Figure 10:**
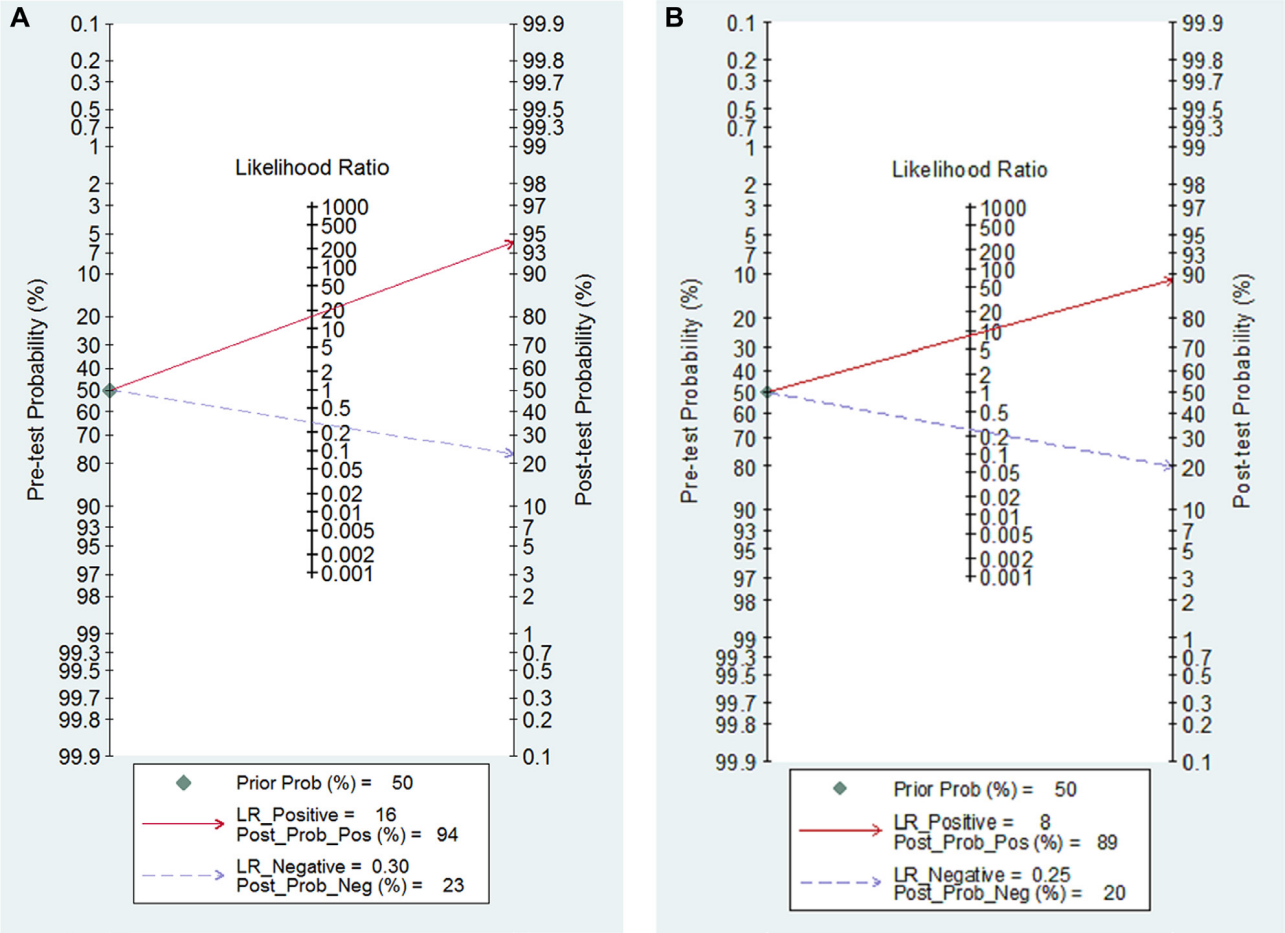
(**A**) Fagan's Nomogram of the SCysC test for diagnosis of eGFR. (**B)** Fagan's Nomogram of the SCr test for diagnosis of eGFR.

SROC curves of the diagnostic value of GFR via SCr and SCysC are shown in Supplementary Figure 6, Supplementary Figure 7. SROC curve of the diagnostic value of GFR via cystatin C and creatinine were shown in Figure 9. The pattern of SCr and SCysC displayed the non-scatter ‘shoulder arm’ shape, indicating a low possibility of threshold effects in the adopted literature. There was no significant difference when comparing the AUC of SCr and SCysC because the confidence intervals overlap (AUC SCr = 0.92 (95% CI: 0.89–0.94), AUC SCysC = 0.93 (95% CI: 0.91–0.95). Posttest probability was calculated with a presumed pretest probability of 50% via Fagan's plot. We described diagnostic values using Fagan's nomogram, which were SCysC and SCr for eGFR (Figure 10A, 10B). When 50% was selected as the pre-test probability, the data showed the pos *t*-test probability increased to 94% for the diagnostic value of SCysC, while the pos*t*-test probability increased only to 89% for the diagnostic value of SCr, indicating that the diagnostic value of SCysC is better than that of SCr.

## DISCUSSION

SCr, SCysC and endogenous creatinine clearance rates were utilized as endogenous indicators to estimate GFR in the clinical setting. In the estimation of GFR, ideal endogenous blood substances incorporate the following properties: release into the blood stream at a steady speed, free filtration by the glomerulus, no reabsorption or secretion by the renal tubules, and removal through the kidneys [28]. Most research mentions many factors that effect the generation of SCr, including age, gender, muscle mass, pharmaceutical or other drug use, among others. In addition, until kidney function loss has reached 50%, a typical concentration of SCr may be seen due to tubular secretion and additional clearance through the viscera [29].

SCr, urine creatinine, serum albumin or SCysC are most often used in eGFR assays [30]. GFR may be calculated via many formulas, and all of these assays and equations were found in the included studies. However, as no single method has been validated in a large human population of CKD patients, finding the best method to assess renal function in CKD patients that can be carried out in routine practice is a problem that needs to be addressed. SCr is suggested to be the typical indicator for renal injury assessment, and plays a significant clinical role in the evaluation of GFR in patients with chronic kidney disease. Additionally, about five to ten percent of discharged creatinine comes from distal tubule discharge, which more or less increases in answer to reduced GFR, making it difficult to accurately identify tiny and mild changes in GFR [31]. Drug treatments such as trimethoprim, cimetidine, cefoxitin that block the distal tubule from releasing creatinine can also enhance the SCr level, leading to increased bias in the estimation of GFR [32]. Normally, in kidney, the glomerulus filters creatinine and the tubule secrets creatinine. However, the secretion of the creatinine can be neglected when the GFR is significantly high [42]. A reported disadvantage of using creatinine is that nearly 50% of the total kidney creatinine excretion is due to proximal tubular secretion, thereby affecting its accuracy in estimating kidney function [62].

Unlike SCr, SCysC is filtered and reabsorbed in the proximal tubules freely, is not secreted by the tubules, and does not rely on sex, race, muscle mass or age. The concentration of SCysC is found to be stable within certain inflammatory conditions and in other disorders of metabolism. And it is possible to SCysC without a well documented limitation to the patients with extreme changes in weight, muscle mass. SCysC rises earlier than SCr when GFR declines, suggesting it could serve as a marker and have further benefits in detecting early renal dysfunction. SCysC has been shown to be better than SCr in GFR assessment, and its usefulness has been demonstrated in patients with almost regular kidney function. In previous studies within the general population and also in older people, SCysC has been shown to be an ideal predictor of death rate and major cardiovascular events, and has been reported to be better than SCr alone. Peralta et al. [31] studied 11,909 participants and found that SCysC levels in those cases may be useful for evaluating CKD individuals with high risk for complications. Including SCysC could enhance death risk forecast by phases of kidney function relative to SCr. The investigators found that comparison of associations using SCysC in estimating GFR can be used to indicate risk of hip fracture in older men. SCysC is produced by nucleated cells in all human beings and its concentration is not influenced by sex, age, dietary habit, swelling, etc. Hence, SCysC has no correlation with pathophysiological states other than GFR. This makes its expression an ideal endogenous marker in estimating GFR changes, and improves the accuracy of early diagnosis for kidney function. Therefore, SCysC provides reliable renal dysfunction risk prediction [33, 34].

A previous meta-analysis written by Shlipak et al. [35] demonstrated that it is possible that rearranging assessment of kidney function to include SCysC improved forecasts of the following: cardiovascular disease, renal morbidity, and mortality. Research by Coll et al. [36] showed that levels of SCr were raised in over 92.1% of patients that had reduced renal function in comparison with SCysC levels, which increased in every patient.

CKD patients or transplant patients should be categorized into stages based on their GFR. This has been suggested by the National Kidney Foundation Disease Outcomes Quality Initiative (K/DOQI) and by Kidney Disease Improving Global Outcomes (KDIGO) [37–40]. Risk of Progression in CKD is usually defined as impaired or decreased kidney function, with a GFR of less than 60 ml/min per 1.73 m^2^ for 3 months or longer, regardless of cause [37]. The results of our meta-analysis show that by SCysC-based estimates of the GFR, we may identify more latent CKD patients when cut-off values are 60 (ml/min/1.73 m^2^), which is most important for secondary prevention of CKD progression. Therefore, we recommend that Cystatin C-based estimates of the GFR should universally be introduced into daily clinical practice or used as endpoints in clinical trials.

However, method limitations must be considered. Unpublished reports could not be identified, which might have biased our results. The aim of the present clinical research was to find an early, sensitive, specific indicator of GFR. In this research, we identified 21 articles to study and systematically performed a meta-analysis to determine the diagnostic value of GFR. The results demonstrated that the DORs of SCysC and SCr have a good correlation with GFR. Based on the forest plots for the degree of SEN and SPE, the pooled effect of the SCysC and SCr values have apparent heterogeneity. Therefore, the likelihood ratio of SCysC has a stronger ability to judge kidney injury and exclude diagnostic effectiveness.

## CONCLUSIONS

There is a significant association of eGFR with SCysC and SCr. Diagnostic accuracy for reduced renal function favors SCysC. The confidence intervals for the grouped sensitivity and specificity for SCr and SCysC intersect. However, SCysC was more sensitive for the estimation of GFR than SCr when the GFR cut-off values are set as 60 (ml/min/1.73 m^2^). Estimating GFR based on SCysC concentration has not been studied extensively, but seems to be a promising method for evaluating the renal function of CKD patients. There is an urgent need for a wholesome prospective study using standardized creatinine assays, an appropriate gold standard and a population that factually represents the total population.

## SUPPLEMENTARY MATERIALS FIGURES AND TABLES


