# Supplementary material for: The diagnostic value of serum creatinine and cystatin c in evaluating glomerular filtration rate in patients with chronic kidney disease: a systematic literature review and meta-analysis
Source: Oncotarget. 2017 Aug 16;8(42):72985–99. doi: 10.18632/oncotarget.20271 (PMC5641185; doi:10.18632/oncotarget.20271)
Supplement: Supplementary file 1 [file oncotarget-08-72985-s001.pdf]

The diagnostic value of serum creatinine and cystatin c in evaluating glomerular filtration rate in patients with chronic kidney disease: a systematic literature review and meta-analysis

SUPPLEMENTARY MATERIALS

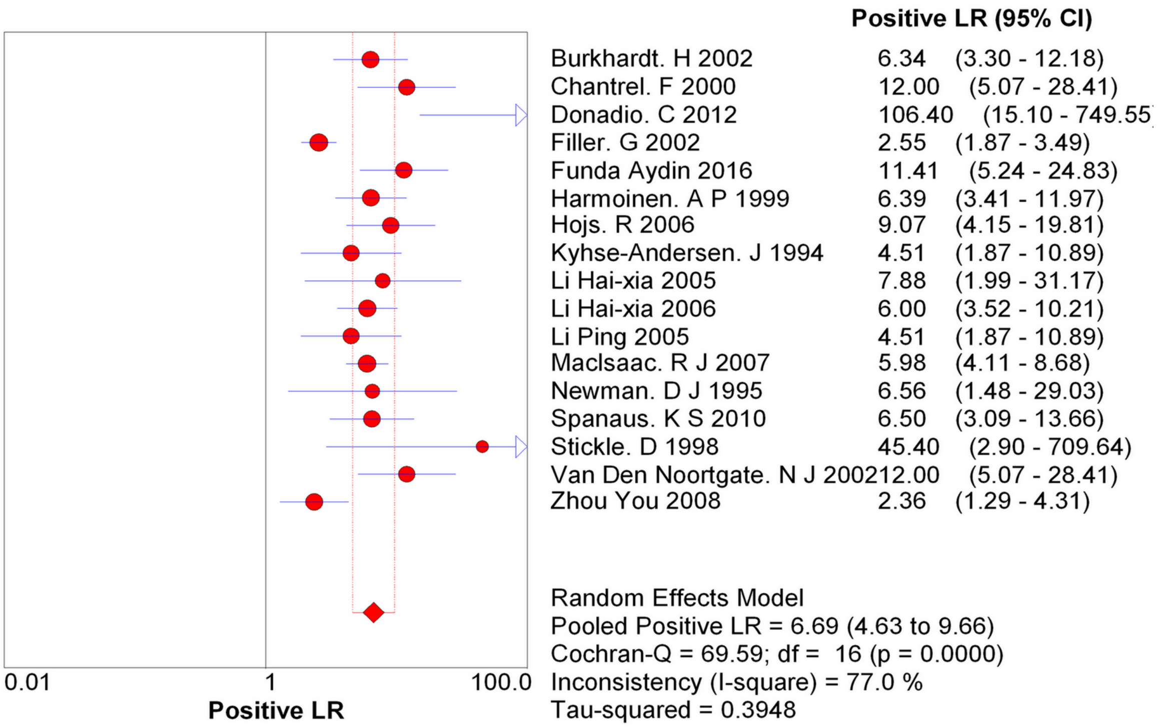

Supplementary Figure 1: The forest plot of positive LR for SCr in evaluating eGFR.

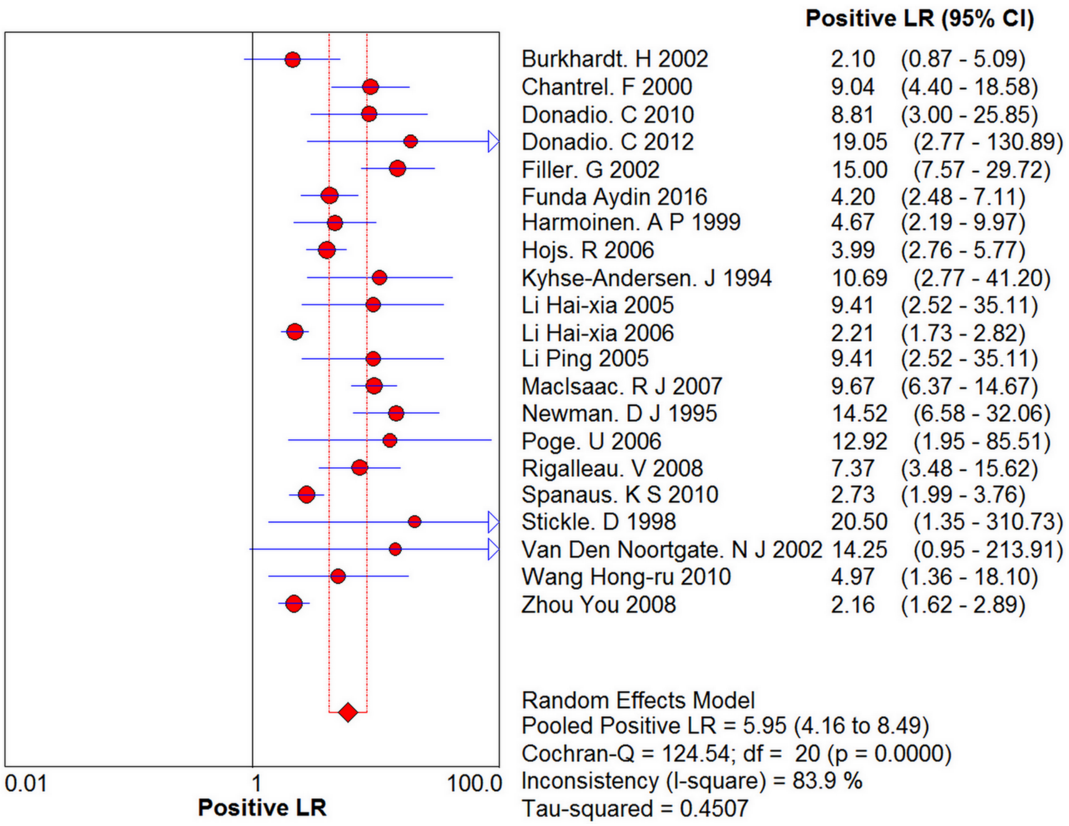

Supplementary Figure 2: The forest plot of positive LR for SCysC in evaluating eGFR.

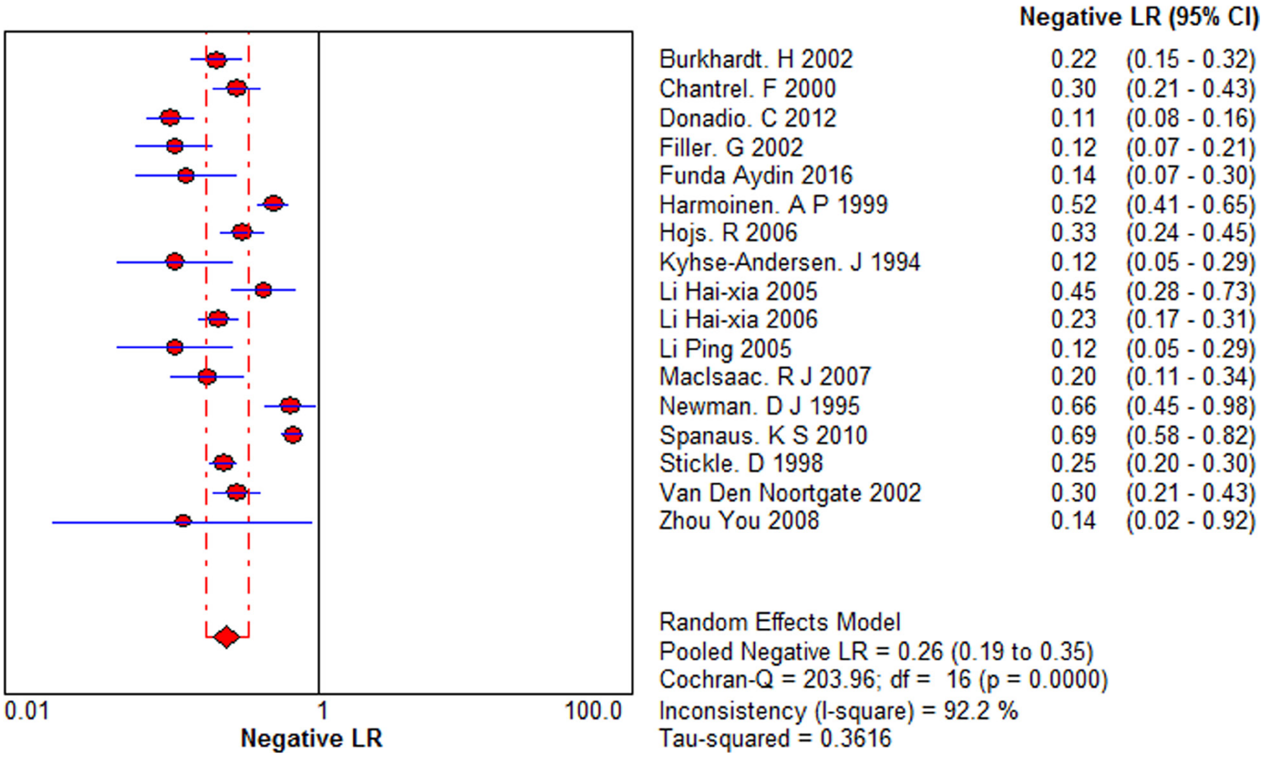

Supplementary Figure 3: The forest plot of Negative LR for SCr in evaluating eGFR.

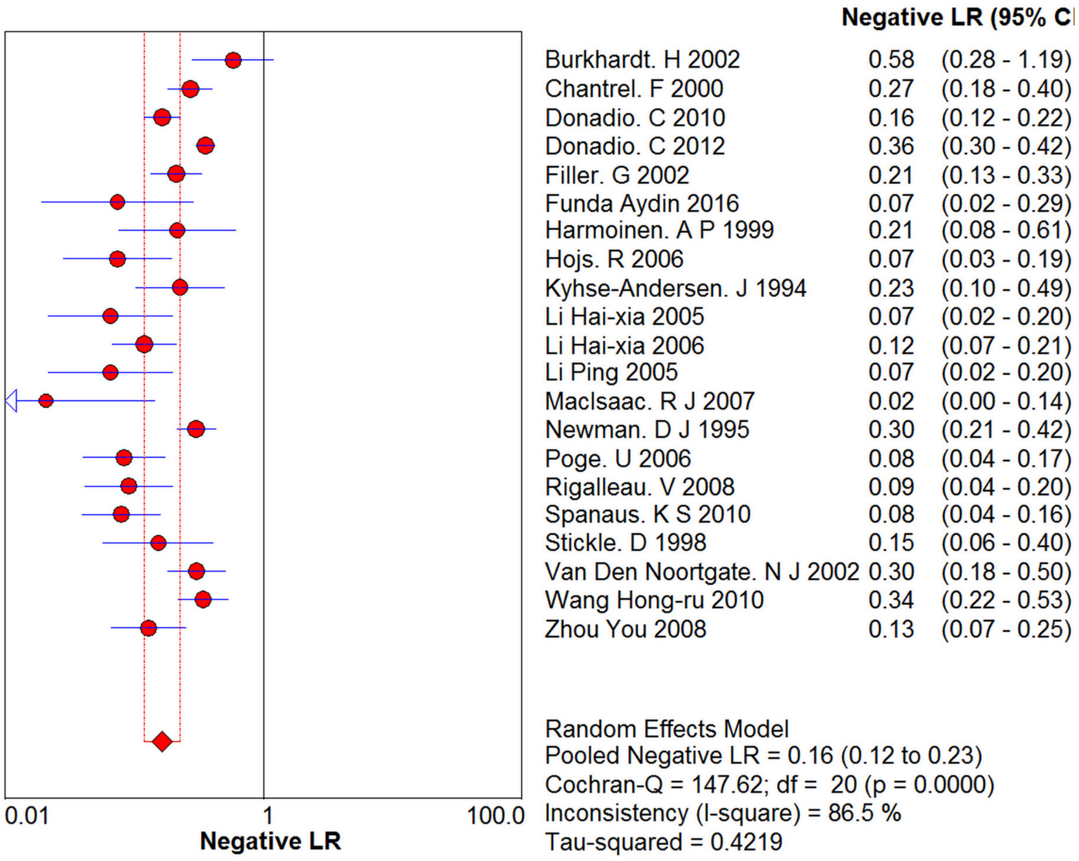

Supplementary Figure 4: The forest plot of Negative LR for SCysC in evaluating eGFR.

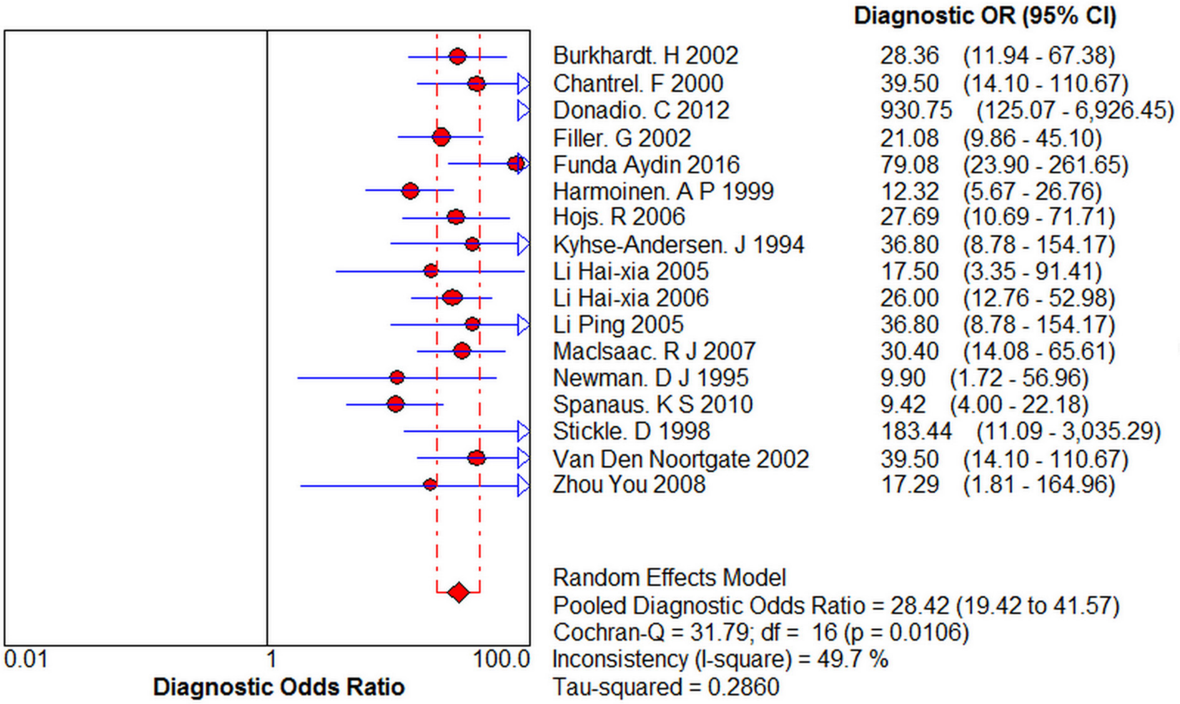

Supplementary Figure 5: The forest plot of DOR for SCr in evaluating eGFR.

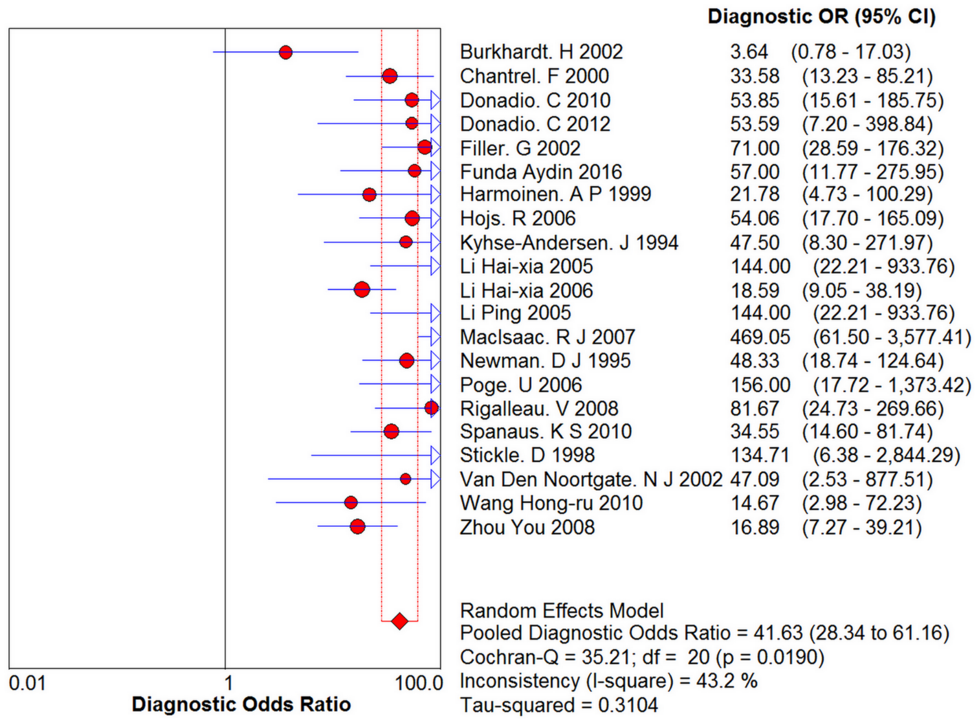

Supplementary Figure 6: The forest plot of DOR for CysC in evaluating eGFR.

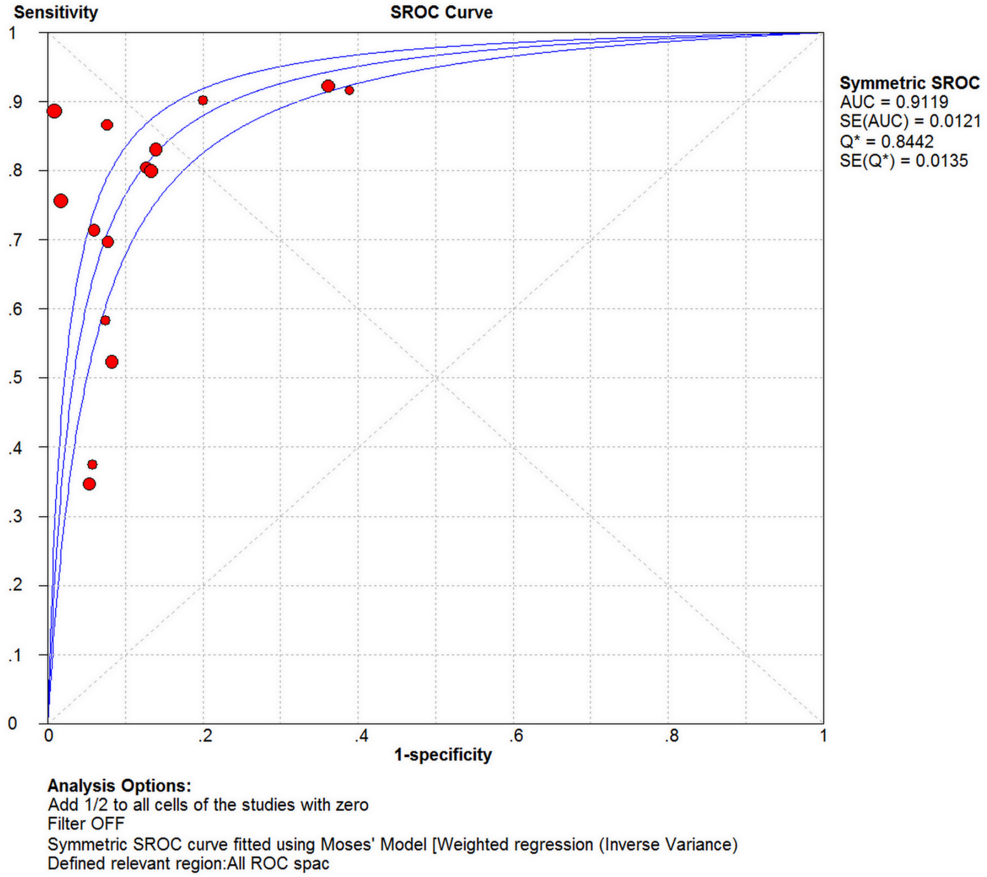

Supplementary Figure 7: The SROC Curve for SCr in evaluating eGFR.

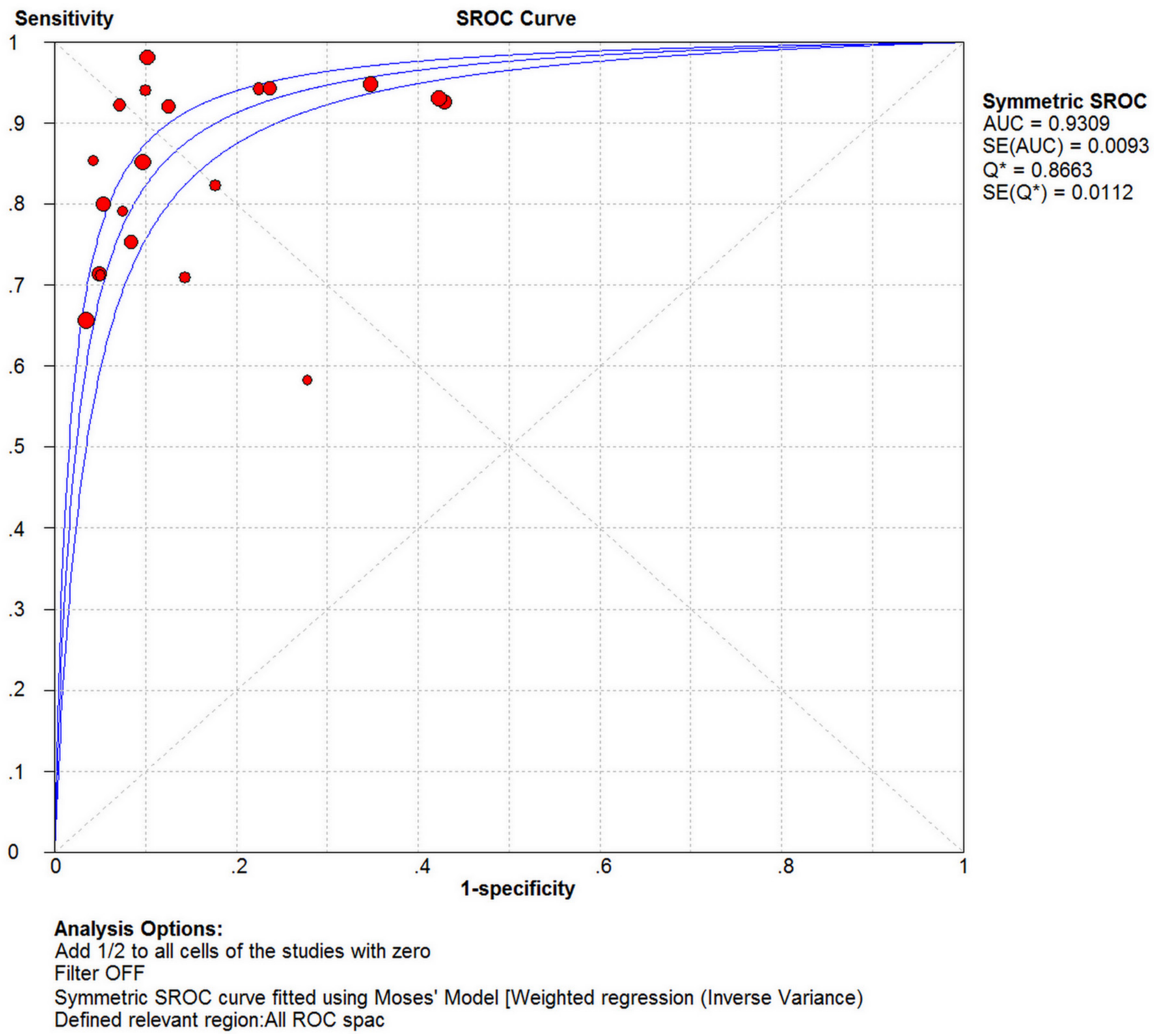

Supplementary Figure 8: The SROC Curve for SCysC in evaluating eGFR.

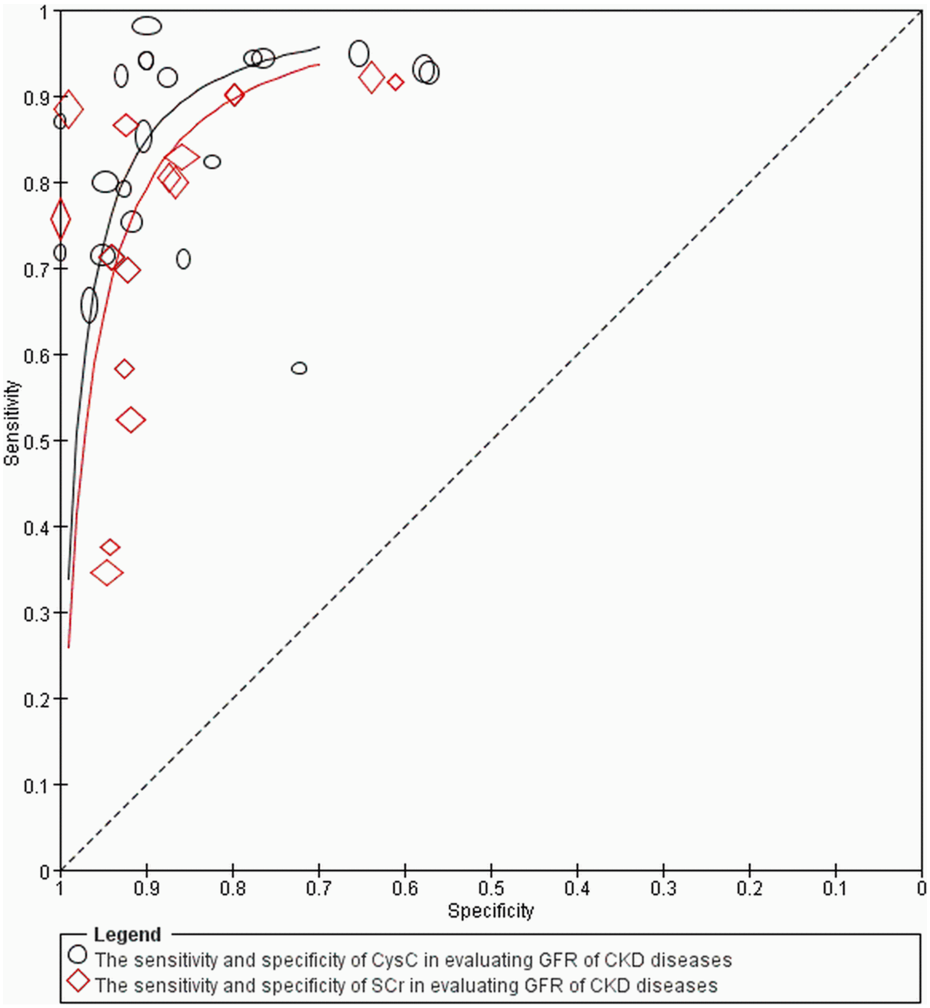

Supplementary Figure 9: The SROC Curve for SCysC and SCr comparison in evaluating eGFR.

Supplementary Table 1: The search strategy for diagnostic value of SCr and SCysC in evaluating GFR in patients with CKD: a systematic literature review and meta. See Supplementary_Table_1

Supplementary Table 2: PRISMA 2009 Checklist. See Supplementary_Table_2
